# Study of Normally-Off AlGaN/GaN HEMT with Microfield Plate for Improvement of Breakdown Voltage

**DOI:** 10.3390/mi12111318

**Published:** 2021-10-27

**Authors:** Xiaoyu Xia, Zhiyou Guo, Huiqing Sun

**Affiliations:** Institute of Semiconductors Science and Technology, South China Normal University, Guangzhou 510631, China; 2019022819@m.scnu.edu.cn (X.X.); sunhq@scnu.edu.cn (H.S.)

**Keywords:** field plate, GaN HEMT, breakdown voltage, normally-off

## Abstract

In this paper, we introduce a new type of AlGaN/GaN high electron mobility transistor (HEMT) with microfield plate (FP). We use Silvaco-ATLAS two-dimensional numerical simulation to calculate the performance of conventional HEMT and HEMT with micro-FP and analyze its principle. By studying a new charge balance method provided by HEMTs and micro-FPs, the physical mechanism of FP adjusting the HEMT potential distribution and channel electric field distribution is analyzed. The new FP structure consists of a drain field plate (D-FP), a source field plate (S-FP) and several micro-gate field plates (G-FP) to improve the output characteristics of HEMTs. By adjusting the distribution of potential and channel electric field, a wider and more uniform channel electric field can be obtained, and the breakdown voltage can be increased to 1278 V. Although the on-resistance of the HEMT is slightly increased to 5.24 Ω mm, it is still lower than other reference values. These results may open up a new and effective method for manufacturing high-power devices for power electronics applications.

## 1. Introduction

GaN-based high electron mobility transistors (HEMTs) have shown great potential for applications in microwave power amplifier and power conversion applications due to their fast switching speed, low switching loss, high saturation electron velocity, and high breakdown voltage (V${}_{br}$) [1,2,3,4,5]. Most of the GaN HEMTs reported are usually in the on state, taking advantage of the inherent high sheet carrier density inherent in the built-in polarized electric field. However, this type of normally open HEMT is not suitable for practical power switching applications where safe operation is the main concern. In this paper, we study normally-off GaN HEMTs [6]. Normally-off operation is usually critical for power electronics applications; thus, a variety of structures are used to implement enhancement-mode transistors, such as gate recess, fluorine incorporation, p-GaN gate, etc. The p-GaN gate structure is widely studied and applied because of its stable operation and good realization of the normally-off function of the device [7].

In addition, many studies have shown that the electric field distribution in HEMT has a decisive influence on equipment performance and equipment reliability. Therefore, electric field optimization is important for HEMTs. Field plate (FP) technology is considered one of the technologies that significantly improves the power performance of HEMT. Using the field plate technology, the depletion region can be effectively expanded, and the single-peak electric field can be replaced by multiple peaks, making the electric field distribution more uniform. Thus, the breakdown voltage performance and current collapse effects can be improved. FP structure does not increase the on-resistance of the HEMT [7]. So far, many types of research on GaN-based HEMT with different FP structures have been reported [8,9,10]. FP technology plays a great role in experiments and chip production. F. Zeng et al. designed and fabricated devices with S-FP and G-FP. It shows that the length of the FP is a key factor to improve the V${}_{br}$ performance, but an excessively long field plate may cause additional current leakage paths [7]. W. SAITO et al. fabricated high V${}_{br}$ AlGaN/GaN HEMT with the FP structure. In this study, 600 V AlGaN/GaN HEMT is experimentally demonstrated using the FP structure, and the results reveal a sufficient V${}_{br}$ and an ultralow on-state resistance [9]. H. Xing et al. fabricated AlGaN/GaN HEMT with multiple FP. With the FP structure, the device showed a V${}_{br}$ as high as 900 V [11]. J. Wong et al. fabricated GaN HEMT with novel asymmetric slant FP; the V${}_{br}$ of the HEMT reached 146 V [12]. Among them, increasing the V${}_{br}$ without increasing the device size is the research focus.

In order to solve the above problems, in this paper, we propose a microfield plate technology. First, the field technology provides a new way of charge balance, and second, the field plate technology has obvious optimization effects on the electric field. Therefore, this field plate technology can solve the existing problems and realize the electric field optimization of GaN-HEMT. This technology increases the breakdown voltage and reduces on-resistance of the device.

## 2. Device Structure and Simulation Model

O. Hilt et al. designed and analyzed a p-GaN gate GaN HEMT with an AlGaN buffer [13]. Compared with the standard GaN buffer HEMT, the P-GaN gate GaN HEMT with AlGaN buffer will yield a higher breakdown voltage, resulting in an excellent V${}_{br}$- to- R${}_{ON}$ ratio. In this paper, we use the P-GaN gate GaN HEMT with an AlGaN buffer as the conventional HEMT structure (without FP structure). The results of this paper are calibrated by previous experimental results.

**Figure 1:**
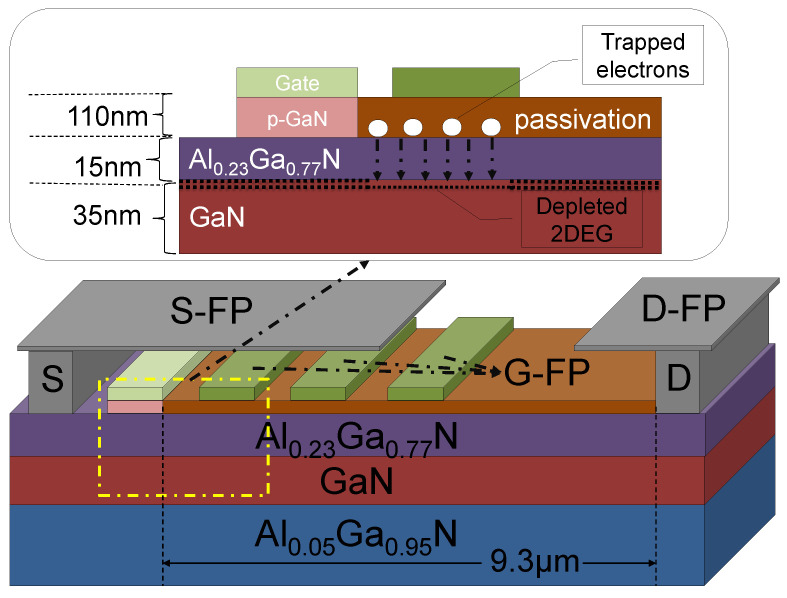
Device structure of high electron mobility transistor (HEMT) with the microfield plate (micro-FP).

**Figure 2:**
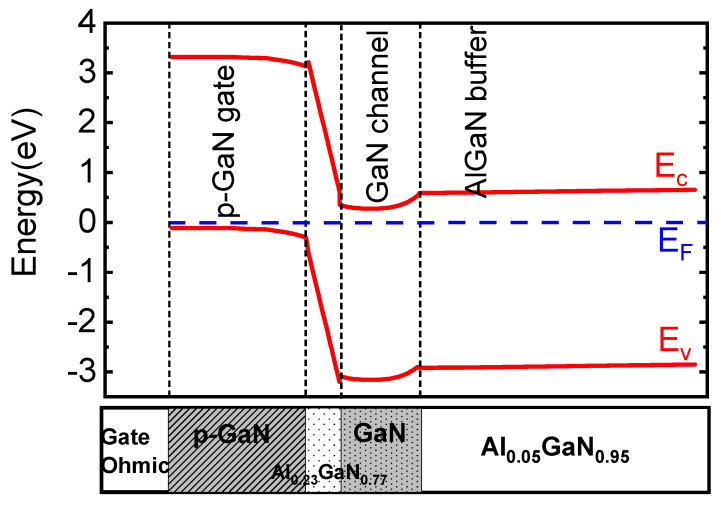
Calculated band diagram of the HEMT with the micro-FP (G−FP=9) under a gate bias of 0 V.

Figure 1 shows the HEMT with a micro-FP structure. The HEMT with a micro-FP structure is different from a conventional HEMT only in that the HEMT with the micro-FP has a field plate structure. The two devices have the same buffer layer, channel layer, barrier layer, and cap layer. From bottom to top, a 2 µm Al${}_{x}$Ga${}_{1-x}$N (x = 0.05) buffer layer, a 35 nm GaN channel layer, and a 15 nm Al${}_{y}$Ga${}_{1-y}$N (y= 0.23) barrier layer were deposited on Si substrates. The gate is composed of Mg-doped GaN with an Ohmic contact for bias. The carrier concentration of p-GaN is $3\times 10^{17}$ cm${}^{-3}$. The p-GaN raises the potential at the channel, thereby realizing normally-off operation, as shown in the conduction band diagram in Figure 2. The polarized charge at the interface between the AlGaN buffer and the GaN channel acts as a virtual p-type doping and supports the p-GaN gate to drag the potential well of the transistor channel out of the Fermi level [13]. The microfield plate structure is composed of a drain field plate (D-FP), a source field plate (S-FP), and several microgate field plates (G-FP). The source–gate distance is 400 nm and the gate–drain distance is 9.3 µm. The length of the D-FP is 3.4 µm, and the G-FPs are evenly distributed in the range of −1 µm to 2.4 µm. Each field plate width is 0.2 µm. Additionally, the lengths of the S-FP and D-FP are 4 µm and 1.4 µm.

The manipulation of FP on the electric field depends on the layout of the entire device, and the thickness, doping concentration, and dielectric constants of each layer will affect the distribution of the electric field. Therefore, the design and optimization of the FP rely on engineering experiments or TCAD. This device is simulated with Silvaco-ATLAS. The polarization effect needs to be considered at the interface between the AlGaN barrier layer and the GaN channel layer to ensure the generation of 2DEG. The polarization also applies to the p-GaN layer and the AlGaN barrier layer to deplete 2DEG under the cap layer. A Shockley–Read–Hall model and a Fermi–Dirac model were used in this work. Select the nitride field-specific mobility model by specifying GANSAT.N and GANSAT.P on the mobility statement. Additionally, the Albrecht model was chosen as the low-field mobility model. Selberherr’s impact ionization model is used for calculating V${}_{br}$. The source and drain contacts are Schottky contacts, and the gate is Ohmic contact [14,15,16,17].

## 3. Simulation Results and Discussion

**Figure 3:**
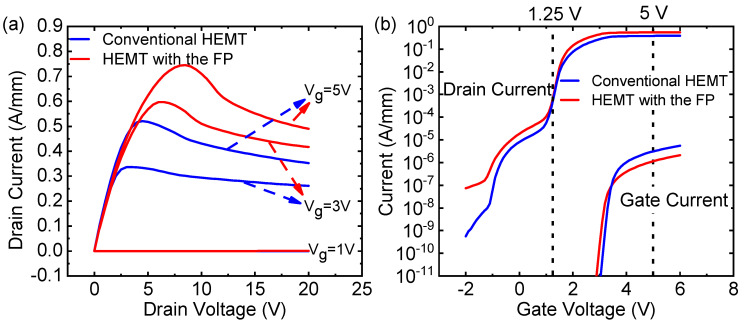
(**a**) Output and (**b**) transfer characteristics of conventional HEMT and HEMT with the micro-FP (G−FP=9).

Figure 3a shows the output characteristics of conventional HEMT and HEMT with the micro-FP (G-FP = 9). When $V_{g}=+5$ V, for the conventional HEMT, the maximum drain current (I${}_{d}$) is 521.24 mA/mm, and the on-resistance is 4.76 Ω mm, whereas for the HEMT with the micro-FP, the current value is increased to 744.9 mA/mm, and the on-resistance is 5.24 Ω mm. Compared with the conventional HEMT, the drain current of the HEMT with the micro-FP is significantly increased, and the on-resistance is slightly increased.

Figure 3b shows the transfer characteristic of conventional HEMT and HEMT with the micro-FP (G-FP = 9). The threshold voltage (V${}_{th}$) is 1.25 V for conventional HEMT, the same as HEMT with the micro-FP. For the two HEMT, the subthreshold leakage current is immediately and greatly reduced below the V${}_{th}$. In the on-state ($V_{g}=+5$ V), the gate current of the conventional HEMT and the HEMT with the micro-FP are 3 µA/mm and 1.15 µA/mm, respectively. However, the drain current of the conventional HEMT and the HEMT with the micro-FP are 0.385 A/mm and 0.551 A/mm, respectively. The gate current is more than five orders of magnitude below the drain current. It can be seen that the field plate structure provides a higher drain current and a lower gate current. However, the on-resistance increases slightly. The AlGaN/GaN HEMT structure causes the electric field to be the strongest near the drain-edge of the gate, which will trap a part of the surface charge, and the impurities in the passivation layer on the device surface will temporarily trap these charges, as shown in Figure 1 inset. When the device is in the on state, the trapped charge will act as a “virtual gate” and consume 2DEG, which will cause the current to collapse and increasing the on-resistance [18]. Due to the micro-G-FP, the electric field at the edge of the gate is homogenized. The trapped electrons are reduced, reducing the loss of 2DEG [19].

**Figure 4:**
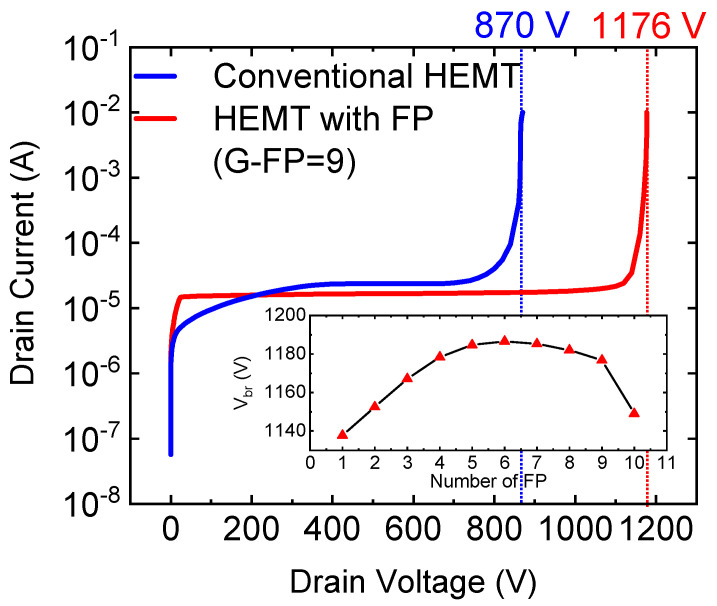
Breakdown characteristics of conventional HEMT and HEMT with the micro-FP.

Breakdown characteristics of conventional HEMT and HEMT with the micro-FP are shown in Figure 4. V${}_{br}$ is defined as the drain voltage when the drain current reaches 0.01 A/mm. The V${}_{br}$ of the conventional HEMT is 870 V. However, the V${}_{br}$ of HEMT with the new FP (G-FP = 9) is 1176 *V*. This indicates that the field plate structure greatly improves the breakdown performance of the device. It can be clearly seen from the Figure 4 insert that the number of G-FP has little effect on the breakdown voltage, especially when the number of 4 ≤ G-FP ≤ 9, V${}_{br}$ is almost the same.

**Figure 5:**
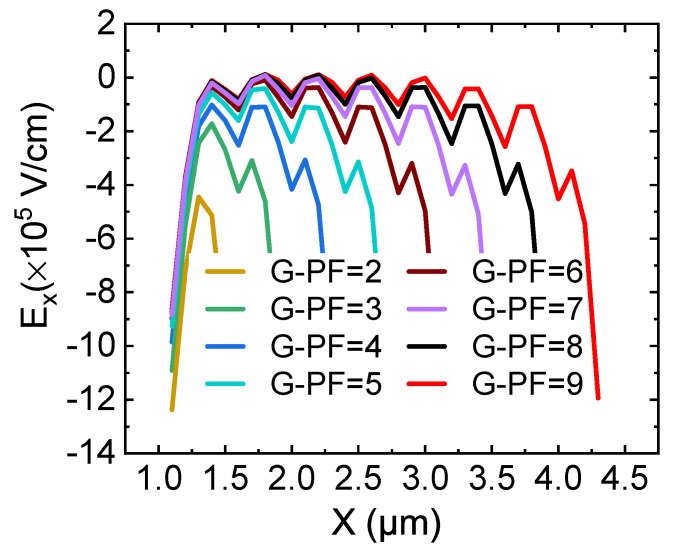
The surface lateral electric field distribution of HEMT with different number of G-FPs under breakdown conditions.

Zhang, Guo et al. found in previous research that a field plate technology can provide a new charge balance effect for lateral power devices [20], and this field plate technology is also suitable for GaN HEMT. Figure 5 shows the surface lateral electric field distribution of HEMT with a different number of G-FPs under breakdown conditions. Since G-FP is periodically in the lateral direction, the electric field distribution along the lateral direction (E${}_{x}$) fluctuates periodically with the number of G-FPs. As the number of G-FPs increases, the fluctuation amplitude and the nonuniformity of E${}_{x}$ distribution decreases. Although the V${}_{br}$ decreases slightly with the increase of G-FPs, when G-FP = 9 the maximum fluctuation of E${}_{x}$ is small. At this point, it can be considered that the distribution of E${}_{x}$ in the lateral direction is uniform. It shows that for the HEMT with G-FP = 9, the charge balance effect is achieved in the entire drift region.

**Figure 6:**
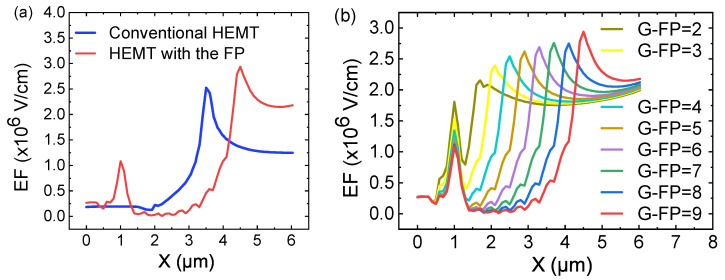
Electric field distribution of the channel region (y=0.02 µm) of (**a**) conventional HEMT and HEMT with the micro-FP (G-FP = 9) (**b**) micro-FP HEMT with different number of G-FP.

Figure 6a shows the electric field (EF) distribution of the channel region (y=0.02 µm) of conventional HEMT and the of HEMT with the micro-FP (G-FP = 9). It can be clearly seen that there are new electric field peaks under the S-FP, G-FP, and D-FP, and there are small peaks under the G-FP region. Compared with the conventional HEMT, the HEMT with the new FP channel has a wider electric field distribution, which also explains the higher V${}_{br}$ of the HEMT with the micro-FP. Figure 6b shows the channel region electric field distribution of HEMT with the micro-FP when the G-FP is 2 to 9. It can be seen from Figure 6b that as the number of G-FP increases, the small peak corresponding to the channel region also increases, and the electric field peaks move to the right. When the G-FP ≥ 4, the electric field distributions are almost the same width. The obtained results are in agreement with Figure 4.

**Figure 7:**
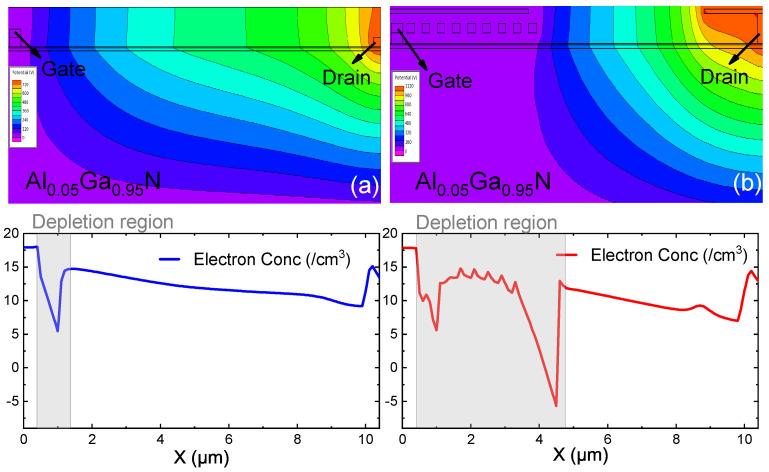
Potential distributions and electron concentrantions of (**a**) conventional HEMT (**b**) HEMT with the micro-FP (G-FP = 9). The value of the electron concentrantions is in the log scale.

From the physical point of view, the modulation effect of the micro-FP structure on the device is studied, and the potential distributions at off-state corresponding to the two HEMT devices, as shown in Figure 7. For conventional HEMT, when the bias increases, the 2DEG near the drain-side gate edge begins to be depleted, thus forming a short depletion region. The potential lines gather on the drain side of the gate, which is why an electric field peak is formed here. For HEMT with the micro-FP, the electron concentration is lower near the drain-side gate edge, indicating a longer depletion region. The potential distribution is more uniform, and three areas of high potential lines density are formed on the edge of the G-FP, the drain, and the edge of the D-FP. It shows the excellent and effective modulation of the potential by the novel FP. Therefore, a more uniform electric field distribution and a higher V${}_{br}$ can be obtained in the HEMT with the micro-FP.

**Figure 8:**
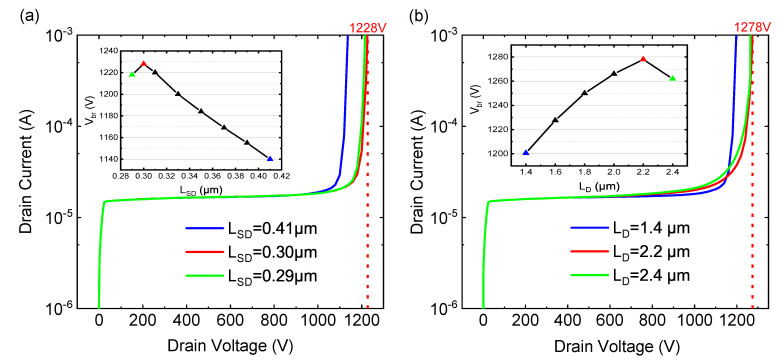
Breakdown characteristics for the variation of (**a**) L${}_{SD}$ (**b**) L${}_{D}$.

In order to study the influence of the distance between the field plates on the breakdown voltage, we study the impact of the distance between the S-FP and the device surface (L${}_{SD}$) on the breakdown voltage (the distance from the D-FP to the device surface is the same) and the influence of the length of D-FP(L${}_{D}$) on the breakdown voltage [7]. The above research is simulated under the condition that the length of S-FP is 2.2 µm and L${}_{D}$ = 1.6 µm. Figure 8a shows breakdown characteristics for the variation of L${}_{SD}$ and L${}_{D}$. When G-FP = 9 V${}_{br}$ gradually increases with the decrease of L${}_{SD}$ and reaches the peak value when L${}_{SD}$ = 0.3 µm, the value is 1228 V. This paper tested a set of V${}_{br}$ with different S-FP lengths; the lengths of S-FP were 2.2 µm, 2.3 µm, 2.4 µm, and 2.5 µm, and a V${}_{br}$ that was almost the same was achieved. The length of S-FP has a negligible influence on V${}_{br}$. Figure 8b shows breakdown characteristics for the variation of L${}_{D}$. When G-FP = 9, the length of S-FP is 2.2 µm and L${}_{SD}$ = 0.3 µm; V${}_{br}$ gradually increases with the increases of L${}_{D}$ and reaches the peak value when L${}_{D}$ = 2.2 µm. The value is 1278 V. It is consistent with the research of F. Zeng, Q. Wang, and S. Lin et al. By optimizing the distance between the field plates, the V${}_{br}$ is increased from 1176 V to 1278 V. Thus, FP technology greatly improves V${}_{br}$ performance.

**Figure 9:**
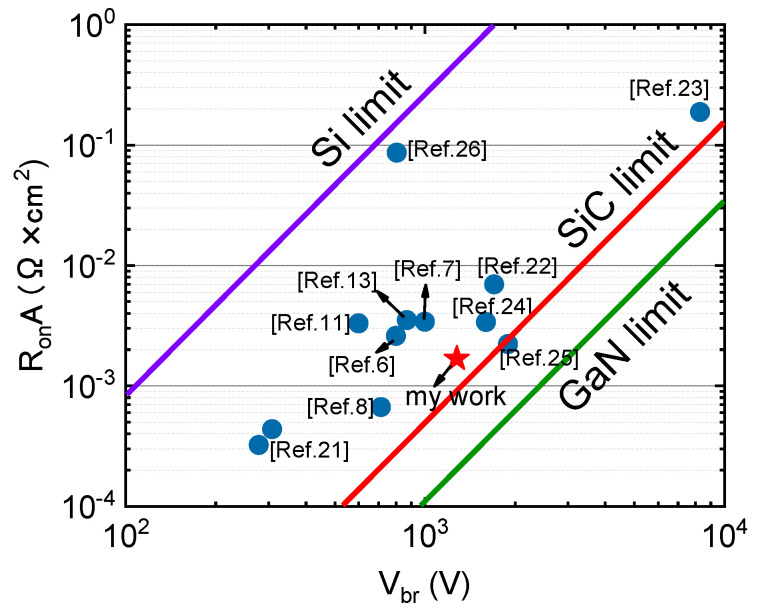
Breakdown voltage versus R${}_{on}$A of the HEMT with the micro-FP, compared with those of GaN-based devices.

The specific on-state resistances R${}_{on}$A versus the V${}_{br}$ of the HEMT with the micro-FP is compared with other reported GaN-based devices in Figure 9 [6,7,8,11,13,21,22,23,24,25,26]. Top performance is demonstrated with R${}_{on}$A = 1.69 mΩ cm${}^{2}$ and V${}_{br}$ = 1278 V in this paper. It can be seen from the figure that this device is not inferior to other reported devices with low on-resistance and high V${}_{br}$. The field plate structure allows the device to operate almost under material limit conditions, thus making full use of the excellent material properties of the AlGaN/GaN system.

## 4. Conclusions

In this paper, we studied the use of microfield plate technology to improve the performance of HEMT through 2D simulation. Results show that the proposed technology can provide a charge balance effect for HEMT, and the HEMT with a micro-FP has better performance than conventional HEMT. This is due to the novel FP structure that can effectively modulate the potential distribution, thereby extending the electric field distribution between the gate and the drain, so that the electric field peaks are concentrated at the edge of the micro-G-FP, the S-FP, and the D-FP. These results are important reference value for the design and actual manufacturing process of HEMT devices, indicating that HEMT with the micro-FP has broad applications in the field of power electronics.

## Data Availability

Not applicable.
